# Evaluation of the Hemocompatibility and Anticancer Potential of Poly(*ε*-Caprolactone) and Poly(3-Hydroxybutyrate) Microcarriers with Encapsulated Chrysin

**DOI:** 10.3390/pharmaceutics13010109

**Published:** 2021-01-16

**Authors:** Eleftherios Halevas, Chrysoula Kokotidou, Elda Zaimai, Alexandra Moschona, Efstratios Lialiaris, Anna Mitraki, Theodore Lialiaris, Anastasia Pantazaki

**Affiliations:** 1 Institute of Biosciences & Applications, National Centre for Scientific Research “Demokritos”, 15310 Athens, Greece; 2 Laboratory of Biochemistry, Department of Chemistry, Aristotle University of Thessaloniki, 54124 Thessaloniki, Greece; eldazaimai@gmail.com; 3 Department of Materials Science and Technology, University of Crete, Voutes Campus, 70013 Heraklion, Greece; chkokoti@hotmail.com (C.K.); mitraki@materials.uoc.gr (A.M.); 4 Institute for Electronic Structure and Laser FORTH, N. Plastira 100, 70013 Heraklion, Greece; 5 Laboratory of Organic Chemistry, Department of Chemical Engineering, Aristotle University of Thessaloniki, 54124 Thessaloniki, Greece; alexmoschona@gmail.com; 6 Laboratory of Natural Resources and Renewable Energies, Chemical Process and Energy Resources Institute, Centre for Research and Technology-Hellas (CERTH), 6th km Harilaou-Thermis, 57001 Thermi, Greece; 7 Laboratory of Genetics, Medical School, Democritus University of Thrace, 68100 Alexandroupolis, Greece; stratoslialiaris1998@hotmail.gr (E.L.); lialiari@med.duth.gr (T.L.)

**Keywords:** chrysin, poly(*ε*-caprolactone) and poly(3-hydroxybutyrate) biodegradable polymers, drug micro-encapsulation and delivery, human blood compatibility, anticancer activity

## Abstract

In this work, novel chrysin-loaded poly(*ε*-caprolactone) and poly(3-hydroxybutyrate) microcarriers were synthesized according to a modified oil-in-water single emulsion/solvent evaporation method, utilizing poly(vinyl alcohol) surfactant as stabilizer and dispersing agent for the emulsification, and were evaluated for their physico-chemical and morphological properties, loading capacity and entrapment efficiency and in vitro release of their load. The findings suggest that the novel micro-formulations possess a spherical and relatively wrinkled structure with sizes ranging between 2.4 and 24.7 µm and a highly negative surface charge with *z*-potential values between (−18.1)–(−14.1) mV. The entrapment efficiency of chrysin in the poly(*ε*-caprolactone) and poly(3-hydroxybutyrate) microcarriers was estimated to be 58.10% and 43.63%, whereas the loading capacity was found to be 3.79% and 15.85%, respectively. The average release percentage of chrysin was estimated to be 23.10% and 18.01%, respectively. The novel micromaterials were further biologically evaluated for their hemolytic activity through hemocompatibility studies over a range of hematological parameters and cytoxicity against the epithelial human breast cancer cell line MDA-MB 231. The poly(*ε*-caprolactone) and poly(3-hydroxybutyrate) microcarriers reached an IC_50_ value with an encapsulated chrysin content of 149.19 µM and 312.18 µM, respectively, and showed sufficient blood compatibility displaying significantly low (up to 2%) hemolytic percentages at concentrations between 5 and 500 µg·mL^−1^.

## 1. Introduction

In recent years, nano- or micro-sized drug delivery systems have become indispensable tools in the pharmaceutical and medical fields and have been extensively investigated due to the growing demand for the controlled administration of pharmacologically active materials to specific cells, tissues, and organelles [1]. Micro-encapsulation offers several significant advantages in vitro and in vivo, as it is used to maintain the structural integrity of compounds that are normally difficult to administer due to insolubility, reactivity, volatility, and hygroscopicity, and to ensure controlled release and protection from degradation of the encapsulated contents. In the pharmaceutical industry micro-encapsulation is widely applied for the specific oral, transdermal, stomach, colon, and small intestine delivery of drugs. More specifically, it has been reported that the encapsulation of drugs into micro-formulations suitable for oral administration reduces the exposure to the harsh conditions of the upper gastrointestinal tract (GIT). Moreover, micro-encapsulation provides immunoisolation and immunoprotection, important factors for the efficient in vivo delivery and implantation of mammalian cells, and cell and tissue engineering applications [2,3].

For the past few decades, natural or synthetic biodegradable polymers such as polyesters, poly(ortho esters), polyanhydrides, polyphosphazenes, chitosan, hyaluronic acid, alginic acid, etc., either as microcapsules, microspheres, or nanoparticles generated under different synthetic techniques [4,5,6,7,8], have been widely applied as carriers for the controlled delivery of bioactive proteins and peptides, as well as hydrophilic or hydrophobic drugs of varied molecular weights [9,10,11,12,13,14] to specific locations in vivo, disintegrating into biocompatible byproducts through enzyme-catalyzed or chemical hydrolysis [15]. The drug-loaded polymeric formulations maintain the biocompatible, physico-chemical and morphological properties of the carrier [16,17,18] and the therapeutic efficacy of the loaded drug [19,20], presenting controllable biodegradation kinetics [21,22,23] and sufficient thermodynamic compatibility between the biopolymer and the encapsulated compound [24].

**Figure 1:**
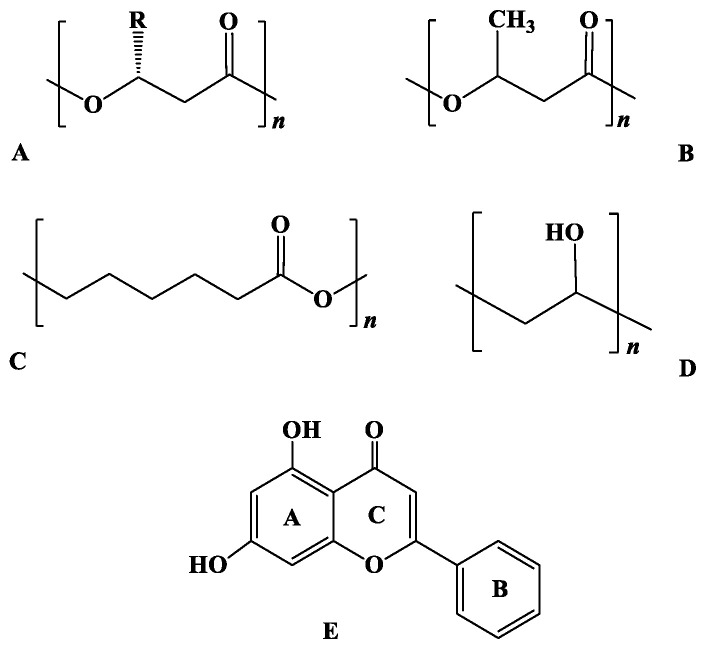
(**A**) General structure of PHAs, (**B**) structure of PHB, (**C**) structure of PCL, (**D**) structure of PVA, (**E**) structure of chrysin.

Two of the best-known groups of biodegradable polymers are polyhydroxyalkanoates (PHAs) (Figure 1A) and poly(*ε*-caprolactones) (PCLs). PHAs are intracellular aliphatic polyesters of diverse hydroxyalkanoate monomers accumulating as energy storage materials by water-insoluble, discrete nano-sized, and optically dense granular inclusions located in the cytoplasm of several bacterial and some archaeal cells, usually in the presence of excess carbon source and under unbalanced growth conditions [1,25]. PHAs are considered exceptional alternatives to various synthetic polymers in a wide range of biomedical applications as drug delivery vehicles or scaffolds in tissue engineering, potentially due to their biodegradability, biocompatibility, and ease of insertion into the human body without having to be removed again and generating significant foreign-body responses to implantation [26]. One of the most widely used PHAs is poly(3-hydroxybutyric acid) (PHB) (Figure 1B). PHB has gained particular attention as drug carrier or scaffold biomaterial because, compared to other biodegradable chemically produced polymers such as poly(lactide-*co*-glycolide) (PLGA), polylactate (PLA), and polyglycolate (PGA), it displays significant advantages, which include remarkable biodegradability and biocompatibility, easier processibility and controllable retarding properties [25]. 

PCL (Figure 1C) is a saturated aliphatic polyester composed by hexanoate repeated units. Based on the range of the weight-average molecular weights, it can be generally described as a semicrystalline material [27]. Due to their ability to be completely degraded by fungal and bacterial enzymes, including esterases and lipases, PCL-based materials are of particular interest in biodegradable material applications [28]. Furthermore, PCL-based formulations, either as blends or as copolymers with synthetic or other biopolymers, due to their remarkable penetrability, nontoxicity, and exceptional biocompatibility, have attracted great attention as controlled drug delivery systems, in cell cultivation and in implants for regenerative medicine as tissue engineering materials [29,30].

However, apart from the significant advantages of both types of biopolymers, their widespread application as drug delivery systems has been restricted by specific shortcomings, such as the slow degradation rate due to their relatively high crystallinity and hydrophobicity. The incorporation of highly hydrophilic, biocompatible, and chemically stable polymers, such as poly(vinyl alcohol) (PVA) (Figure 1D), as stabilizers and dispersing agents with favorable mechanical properties for the emulsification procedure of the biopolymers, results in the generation of formulations with improved hydrophilicity and optimized degradation rates [31].

Flavonoid chrysin (5,7-dihydroxyflavone) (Figure 1E) (Empirical formula: C_15_H_10_O_4_, Molecular weight: 254.24 g·mol^−1^, Melting point: 284–286 °C, Solubility: 0.1 M in NaOH 0.008 g·L^−1^, λ_max_: 348 nm) is present in honey, propolis, and honeycombs and is also a constituent of the blue passion flowers extract [32]. Chrysin (Chr) displays antioxidant, antiallergic, anti-inflammatory [33], and important pharmacological and biochemical properties associated with the prevention of cancer [34], functioning as an inhibitor for cell proliferation and tumor angiogenesis in vivo [35], and tumor cell apoptosis in vitro [36]. However, despite its significant biological properties, the (a) short terminal half-life, (b) quick metabolism, (c) low absorption rate, and (d) poor bioavailability limit its therapeutic efficacy [37]. As a result, several types of formulations have been produced for the efficient encapsulation of chrysin in an effort to overcome limitations arisen through its low aqueous solubility and bioavailability. However, until today, only nanosized formulations of encapsulated chrysin have been reported in the literature utilizing combinations of PLGA/PVA, methoxy poly(ethylene glycol)-β-PCL, PLGA-poly(ethylene glycol), PLGA-poly(ethylene glycol)-PLGA, magnetic SiO_2_/poly(ethylene glycol), or PLGA-poly(ethylene glycol) chemically produced polymers [38,39,40,41,42,43].

Aiming at the development of novel multifunctional pharmaceutical micro-formulations with enhanced bioavailability and therapeutic efficacy involving bioactive flavonoids, we report herein, for the first time, the synthesis, physico-chemical characterization, and biological evaluation of empty and chrysin-loaded PVA-stabilized PCL and PHB microcarriers (MCs) (mentioned henceforth as EPCL/PVAMCs, EPHB/PVAMCs, ChrPCL/PVAMCs, and ChrPHB/PVAMCs, respectively). The newly synthesized micromaterials were compared and evaluated for their suitability as potential MCs with controlled release and optimized solubility and bioavailability of chrysin, and their structural and textural properties were determined by different and complementary physico-chemical characterization techniques. The cytotoxic effect of the novel micro-formulations was evaluated against the epithelial human breast cancer cell line MDA-MB-231, and their hemolytic capacity was determined through human blood compatibility studies over a range of hematological parameters.

## 2. Experimental

### 2.1. Materials

The initial materials used include: Polycaprolactone (PCL) (average mol wt. 45,000), poly[(R)-(3-hydroxybutyric acid)] (PHB) (average mol wt. 10,000), poly(vinyl alcohol) (PVA) (87–90% hydrolyzed, average mol wt 30,000–70,000), chrysin powder (purity 97%), sodium hydroxide pellets (NaOH), phosphate buffered saline pH 7.4 (PBS). These materials were purchased from commercial sources (Sigma, Fluka, St. Louis, MO, USA) and were used without further purification. Ultrapure water, chloroform, dichloromethane, methanol, and dimethyl sulfoxide (DMSO) were used as solvents.

The epithelial human breast cancer cell line MDA-MB-231 was from our cell bank at the Institute of Molecular Biology and Biotechnology (IMBB), FORTH, and was free of mycoplasma contamination. The media/agents for the cell cultures were purchased from Thermo Fisher Scientific (Waltham, MA, USA). The MTT reagent (3-[4,5-dimethylthiazol-2-yl]-2,5-diphenyl-tetrazolium bromide) was bought from Sigma-Aldrich (Darmstadt, Germany).

### 2.2. Methods

#### 2.2.1. Fourier-Transform Infrared Spectrometry

Fourier-transform infrared spectra (FT-IR) were recorded on a Perkin Elmer 1760X FT-infrared spectrometer (Perkin-Elmer, San Francisco, CA, USA). 90 mg of KBr was mixed with 10 mg of each sample by grinding in agate mortar. A disk was made using the obtained powdered mixture under a hydraulic pressure of 600 kg. Subsequently, the FT-IR spectra were recorded between 4000 and 450 cm^−1^, with a spectral resolution of 2 cm^−1^.

#### 2.2.2. Field Emission Scanning Electron Microscopy

The morphology and detailed structural features of the EPCL/PVAMCs, EPHB/PVAMCs, ChrPCL/PVAMCs, and ChrPHB/PVAMCs samples were investigated by field emission scanning electron microscopy (FESEM), using a JEOL JSM-7000F microscope (JEOL, Welwyn Garden City, Hertfordshire, UK). A 10 µL sample (PBS dispersion, diluted 1:10) was deposited on a circular cover glass (immobilized on a double-sided carbon tape) and was air dried overnight. Samples were additionally covered with 10 nm Au/Pd sputtering. The analyses were performed in high vacuum mode in a 15 kV accelerating voltage.

#### 2.2.3. Dynamic Light Scattering

Mean particle size was determined through Dynamic Light Scattering (DLS) using Photon Correlation Spectroscopy (Malvern S4700 PCS System, Malvern Instruments Ltd., Malvern, UK). The analysis was performed at a scattering angle of 90° and at a temperature of 25 °C, using appropriately diluted samples (10 mg of each sample in 50 mL PBS, pH 7.4). Before the measurements, the samples were sonicated for 5 min. For each sample, the mean diameter ± standard deviation (±SD) of six determinations was calculated applying multimodal analysis.

#### 2.2.4. Zeta-Potential

Zeta-potential measurements of EPCL/PVAMCs, EPHB/PVAMCs, ChrPCL/PVAMCs, and ChrPHB/PVAMCs samples were determined by Laser Doppler Anemometry (Malvern Zetasizer IV, Malvern Instruments Ltd., Malvern, UK). All analyses were performed on samples appropriately diluted with 1 mM PBS buffer (adjusted to pH 7.4) in order to maintain constant ionic strength and after sonication for 5 min and subsequent filtration. For each sample, the mean value ±SD of four determinations was established.

#### 2.2.5. High Performance Liquid Chromatography

The determination of entrapment efficiency and loading capacity, and the in vitro release study of chrysin were performed through High Performance Liquid Chromatography (HPLC) at a ThermoFinnigan Spectra HPLC system (San Jose, CA, USA) model UV 6000 LP, equipped with EZChromeElite software, Version 3.1.7, four Q-Grad pumps, a diode array detector (DAD) and a Grace Smart RP C-18 column (250 × 4.6 mm id.; 5 µm particle size). The injection volume was 20 µL, and the wavelength used was 268 nm. The mobile phases were 2% (*v*/*v*) acetic acid in milli-Q H_2_O (eluent A) and 100% acetonitrile (eluent B) with a flow rate of 1 mL·min^−1^. The elution profile was as follows: from 0 min, 100% A; 25 min, 36% A/64% B; 35 min, 25% A/75% B.

### 2.3. Synthesis of Chrysin-Loaded MCs

#### 2.3.1. Synthesis of ChrPCL/PVAMCs

The ChrPCL/PVAMCs were formulated via a modification of the oil-in-water (O/W) single emulsion/solvent evaporation method [39]. More specifically, 500 mg of PCL (average mol wt. 45,000) was dissolved in 100 mL of dichloromethane under continuous stirring at room temperature in a 200 mL closed vessel. Subsequently, a solution of 100 mg of chrysin in 40 mL of a dichloromethane/methanol mixture (3:1, *v*/*v*) was added to the above mixture and left stirred for another 24 h at room temperature. After complete homogenization of the mixtures (organic phase), the O/W emulsion was prepared by the dropwise addition of the organic phase to an aqueous solution of PVA (20 mL, 5% *w*/*v* PVA, 87–90% hydrolyzed, average mol wt 30,000–70,000) (aqueous phase). The emerged mixture was homogenized at 15,000 rpm for 30 min, emulsified by sonication for two 5 min periods interrupted by a 2 min resting period in an ice bath, and left stirred with an overhead propeller under a flow hood at 600 rpm for 12 h for the complete evaporation of the organic solvents. ChrPCL/PVAMCs were collected as a yellow precipitate through centrifugation at 6000 rpm for 30 min. They were washed two times with PBS and centrifuged again at 6000 rpm for 10 min to ensure the removal of non-encapsulated chrysin. Finally, the emerged material was immediately freeze-dried at −35 °C and 0.4 mbar for 72 h and stored at 4 °C until further analysis.

EPCL/PVAMCs were prepared following the same experimental procedure without the addition of chrysin and were used as control samples in the ensuing biological experiments. 

#### 2.3.2. Synthesis of ChrPHB/PVAMCs

The ChrPHB/PVAMCs were formulated based on a similar synthetic procedure, as in the case of ChrPCL/PVAMCs. More specifically, 500 mg of PHB (granule, 5 mm nominal granule size) was dissolved in 100 mL of a chloroform/methanol mixture 4:1 (*v*/*v*) under continuous stirring at room temperature in a 200 mL closed vessel. Subsequently, a solution of 100 mg of chrysin in 40 mL of a chloroform/methanol mixture (3:1, *v*/*v*) was added to the above mixture and left stirred for another 24 h at room temperature. After complete homogenization of the mixtures (organic phase), the O/W emulsion was prepared by the dropwise addition of the organic phase to an aqueous solution of PVA (20 mL, 5% *w*/*v* PVA, 87–90% hydrolyzed, average mol wt 30,000–70,000) (aqueous phase). The mixture was homogenized at 15,000 rpm for 30 min, emulsified by sonication for two 5 min periods interrupted by a 2 min resting period in an ice bath, and left stirred with an overhead propeller under a flow hood at 600 rpm for 12 h for the complete evaporation of the organic solvents. ChrPCL/PVAMCs were collected as a yellow precipitate through centrifugation at 6000 rpm for 30 min. They were washed two times with PBS and centrifuged again at 6000 rpm for 10 min to ensure the removal of non-encapsulated chrysin. Finally, the emerged material was immediately freeze-dried at −35 °C and 0.4 mbar for 72 h and stored at 4 °C until further analysis.

EPHB/PVAMCs were prepared following the same experimental procedure without the addition of chrysin and were used as control samples in the ensuing biological experiments. 

### 2.4. Determination of Chrysin Entrapment Efficiency and Loading Capacity

The determination of the entrapment efficiency and loading capacity of chrysin into the PCL/PVAMCs and PHB/PVAMCs was estimated via HPLC analysis. More specifically, 50 mg of dry ChrPCL/PVAMC or ChrPHB/PVAMC sample was ground and immersed in a 50 mL PTFE beaker, into 20 mL of a DMSO/methanol mixture (1:1, *v*/*v*) and then filtered through a 0.45 µm PTFE-membrane syringe filter. Appropriate dilutions were applied for the HPLC measurements, and the chrysin content was determined according to the following calibration curve:

C_Chr_ = 5 × 10^8^ × peak area − 5 × 10^6^ (R² = 0.998),

where C_Chr_ stands for chrysin concentration in the sample (mg∙mL^−1^) and the peak area is the area of the sample measured at 268 nm.

The entrapment efficiency of chrysin was calculated using the following equation:

Entrapment Efficiency (%) = Chr_i_/Chr_t_ × 100,

where Chr_i_ is the amount of chrysin incorporated into each type of MCs and Chr_t_ is the initially added amount of chrysin.

The loading capacity of chrysin was calculated using the following equation:

Loading Capacity (%) = Chr_i_/W_Chr-loaded MCs_ × 100,

where Chr_i_ is the amount of chrysin incorporated into each type of MCs and W_Chr-loaded MCs_ is the weight of the synthesized chrysin-loaded MCs after freeze drying.

Experiments were carried out in triplicates, and the results were expressed as mean ±SD.

### 2.5. In Vitro Chrysin Release Study

The determination of the chrysin release profile from ChrPCL/PVAMCs and ChrPHB/PVAMCs was performed as follows: 50 mg of each type of chrysin-loaded MCs was ground and immersed into 20 mL of a PBS (pH 7.4, 1% *v*/*v* DMSO) solution [44,45]. The release medium temperature was set at 37 ± 1 ^o^C under continuous stirring at a rate of ca. 250 rpm [46]. Aliquots of 1.5 mL were withdrawn with a syringe at fixed time intervals for analysis followed by appropriate dilutions. Following removal of insoluble solid chrysin-loaded MCs by centrifugation (13,000 rpm, 1 min) and filtration (0.45 µm PTFE-membrane syringe filter), the remaining clear solution was analyzed, and the amount of chrysin released was determined by HPLC with the aid of the aforementioned calibration curve (vide supra). The cumulative release percentages of chrysin were calculated according to the following equation:

Cumulative chrysin release (%) = Chr_RELEASED_/Chr_ENTRAPPED_ × 100

The percentages of the insoluble solid chrysin-loaded MCs are presented in the Supporting Information, Table S1.

Experiments were carried out in triplicates, and the results were expressed as mean ±SD.

### 2.6. Biological Evaluation

#### 2.6.1. Cell Lines and Culture Conditions

Epithelial human breast cancer MDA-MB-231 cells were grown in Dulbecco’s Modified Eagle′s—Medium (DMEM) growth medium (pH 7.4) supplemented with 10% Fetal Bovine Serum (FBS) and 50 µg·mL^−1^ gentamycin at 37 °C in a 5% humidified CO_2_ incubator. 

#### 2.6.2. Cell Viability of Human Breast Cancer Cell Line (MDA-MB-231)

In the present study, the cell viability of MDA-MB-231 breast cancer line was evaluated via the MTT assay. The method relies on the conversion of the yellow 3-(4,5-dimethylthiazol-2-yl)-2,5-diphenyl tetrazolium bromide (MTT) to purple formazan crystals. The reduction of MTT is catalyzed by the mitochondrial dehydrogenase enzyme and is therefore a measure for cell viability. MDA-MB-231 cells were exposed to free chrysin, ChrPCL/PVAMCs, or ChrPHB/PVAMCs in their exponential phase of growth. More specifically, in a 96-well plate (Corning, NY, USA), a number of 1 × 10^4^ cells/well were seeded, and after 24 h (80% confluency) they were treated with different concentrations of ChrPCL/PVAMCs or ChrPHB/PVAMCs ranging between 0–400 µg·mL^−1^. Due to the different loading capacity of chrysin in each type of MCs, free chrysin (dissolved in DMSO) was added accordingly, in equal concentrations (µM), with the encapsulated chrysin in the MCs, as estimated in Section 3.5. The cells were incubated with the chrysin-loaded MCs or with free chrysin for 48 h. After incubation, the medium was replaced with 90 µL of fresh DMEM and 10 µL of MTT (5 mg·mL^−1^) per well. The plate was then incubated for 4 h at 37 °C in a 5% humidified CO_2_ incubator. Subsequently, the content of the wells was carefully removed, and 100 µL of a solution of DMSO/isopropanol in a 1:1 ratio was added to achieve the dissolution of the formazan crystals, and then the plate was incubated for 15 min in 37 °C and 15 min in 4 °C. Finally, a Synergy HTX BioTEK plate reader (with a reference wavelength of 630 nm) was used to determine the absorbance measurement at 545 nm. All the experiments were performed in triplicate.

### 2.7. Blood Sample Collection and Handling

Human blood samples were freshly collected from ten healthy volunteers and divided into tubes containing the anticoagulant agent ethylenediamine tetraacetic acid (EDTA), according to the protocols approved by the National Institute of Health and the Food and Drug Administration.

#### 2.7.1. Blood Profile Analysis

The blood profile analysis was performed using an automatic hematological analyzer Beckman Coulter ACT 5 Diff OV, (Beckman Coulter International S.A., Nyon, Switzerland) for the determination of different hematological parameters, such as red blood cells (RBCs) count (10^12^/µL), hemoglobin (HGB) g·dL^−1^), hematocrit (HCT) (%), mean corpuscular volume (MCV) (in femtoliters, fl), mean corpuscular hemoglobin (MCH) (pg), mean corpuscular hemoglobin concentration (MCHC), red cell distribution width (RDW), white blood cells (WBCs) (10^9^/L), neutrophils (NE) (%), lymphocytes (LY) (%), monocytes (MO) (%), eosinophils (EO) (%), basophils (BA) (%), and platelets (PLTs) (10^9^/L), after exposure to free chrysin, ChrPCL/PVAMCs, or ChrPHB/PVAMCs, and without exposure to any agent. Briefly, the sample preparation was performed as follows: A 100 µL sample of whole blood was added to 900 µL of PBS. Then, either chrysin, ChrPCL/PVAMCs, or ChrPHB/PVAMCs were added to this diluted blood to achieve three concentrations: low, high, and very high (5, 80, and 200 µg·mL^−1^, respectively). The negative control sample used consisted of 100 µL of whole blood diluted with 900 µL PBS. In parallel, 100 µL samples of whole blood were subjected to the same treatment with the addition of EPCL/PVAMCs or EPHB/PVAMCs. The suspensions were incubated at 37 °C for 1 h. All the experiments were performed in triplicate.

#### 2.7.2. Hemocompatibility Studies

The hemocompatibility of free chrysin and the produced micro-formulations was evaluated via the hemolysis assay performed using a biochemical analyzer Konelab 30, Thermo Scientific [47]. The experimental procedure was as follows: Whole blood samples were centrifuged for 10 min at 1500 rpm to remove plasma. The obtained cell pellets were washed three times with sterile PBS (10 mM, pH 7.2) to separate the red blood cells (RBCs) from other blood components, such as the white blood cells, plasma proteins, and excess antibodies, centrifuged and finally re-suspended at 5 mL PBS. The hemolysis assay was performed by adding 100 µL of the RBC suspension to 900 µL of PBS, containing several concentrations (5, 20, 40, 60, 80, 100, 200, 300, 400, 500 µg·mL^−1^) of free chrysin, ChrPCL/PVAMCs, or ChrPHB/PVAMCs. The positive (+) control sample of hemolysis used (100% hemolysis) consisted of 900 µL of ultrapure water and 100 µL of washed RBCs. The negative (−) control sample (0% hemolysis) consisted of 900 µL PBS and 100 µL of washed RBCs. PBS and PBS containing either free chrysin, ChrPCL/PVAMCs, or ChrPHB/PVAMCs were used as blank samples. All samples were incubated for 24 h at 37 °C, under agitation at 120 rpm. After incubation, they were centrifuged at 700 rpm for 5 min, and the absorbance of the supernatant was measured at 541 nm. The % hemolysis was calculated after subtracting the blank values, and by setting the control (+) value as 100% of hemolysis. The absorbance was transformed to hemolysis percentage using the following equation:

$$\mathrm{Percentage}\;\mathrm{of}\;\mathrm{hemolysis}\;\left(\%\right)=\frac{{\mathrm{OD}}_{\mathrm{Sample}}\;-{\;\mathrm{OD}}_{\mathrm{Negative}\;\mathrm{control}}}{{\mathrm{OD}}_{\mathrm{Positive}\;\mathrm{control}}\;-{\;\mathrm{OD}}_{\mathrm{Negative}\;\mathrm{control}}}$$

where OD stands for Optical density.

#### 2.7.3. Statistical Analysis

Data are the mean of at least three independent experiments. The statistical significance of changes in different groups was evaluated by one-way analysis of variance (ANOVA) followed by Student t-tests, using GraphPad Prism 6.0 software (Science Plus Group, Groningen, The Netherlands). For each experiment, data are expressed as the mean ±SD, * *p* ≤ 0.05, ** *p* ≤ 0.01, *** *p* ≤ 0.001, ns (not significant) > 0.05.

## 3. Results and Discussion

### 3.1. Synthesis of Chrysin-Loaded MCs

**Figure 2:**
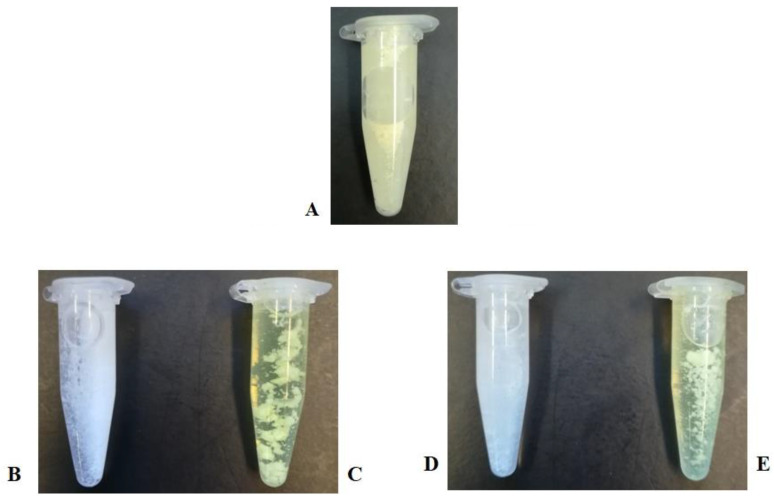
PBS-dispersed (1% *v*/*v* DMSO) (**A**) chrysin, (**B**) EPCL/PVAMCs, (**C**) ChrPCL/PVAMCs, (**D**) EPHB/PVAMCs, and (**E**) ChrPHB/PVAMCs.

The chrysin-loaded PCL/PVA and PHB/PVA MCs were synthesized according to a modified O/W single emulsion/solvent evaporation method which is usually employed for the encapsulation of hydrophobic compounds, such as chrysin. The emulsification step was performed by the addition of the organic phase with the dissolved biopolymer (PCL or PHB) and flavonoid to an aqueous PVA solution, followed by high-speed homogenization, sonication, and subsequent evaporation of the organic solvents. The PVA used was 87–90% hydrolyzed, which is a degree of hydrolysis that ensures the optimum solubility of PVA in water [48]. In general, the addition of a PVA surfactant as a stabilizing and emulsifying agent enhances the stability of the dispersed phase droplets formed during the process of emulsification via the emerging interactions between the hydroxyl groups in its structure with the aqueous phase and the vinyl chain with the organic phase, thus inhibiting microsphere flocculation and coalescence [49,50]. Furthermore, the addition of the highly hydrophilic PVA limits the hydrophobic nature of the produced micro-formulations, promoting the formation of more amphiphilic species [31]. Chrysin is encapsulated within the produced MCs through non-covalent interactions forming molecule-in-molecule assemblies via hydrogen bonds and weak van der Waals forces with the functional groups of the MC hosts [51]. The effective encapsulation aims at enhancing chrysin aqueous solubility and therefore its systemic bioavailability, targeted delivery, circulation time, and therapeutic potential (Figure 2).

### 3.2. FT-IR Spectroscopy

**Figure 3:**
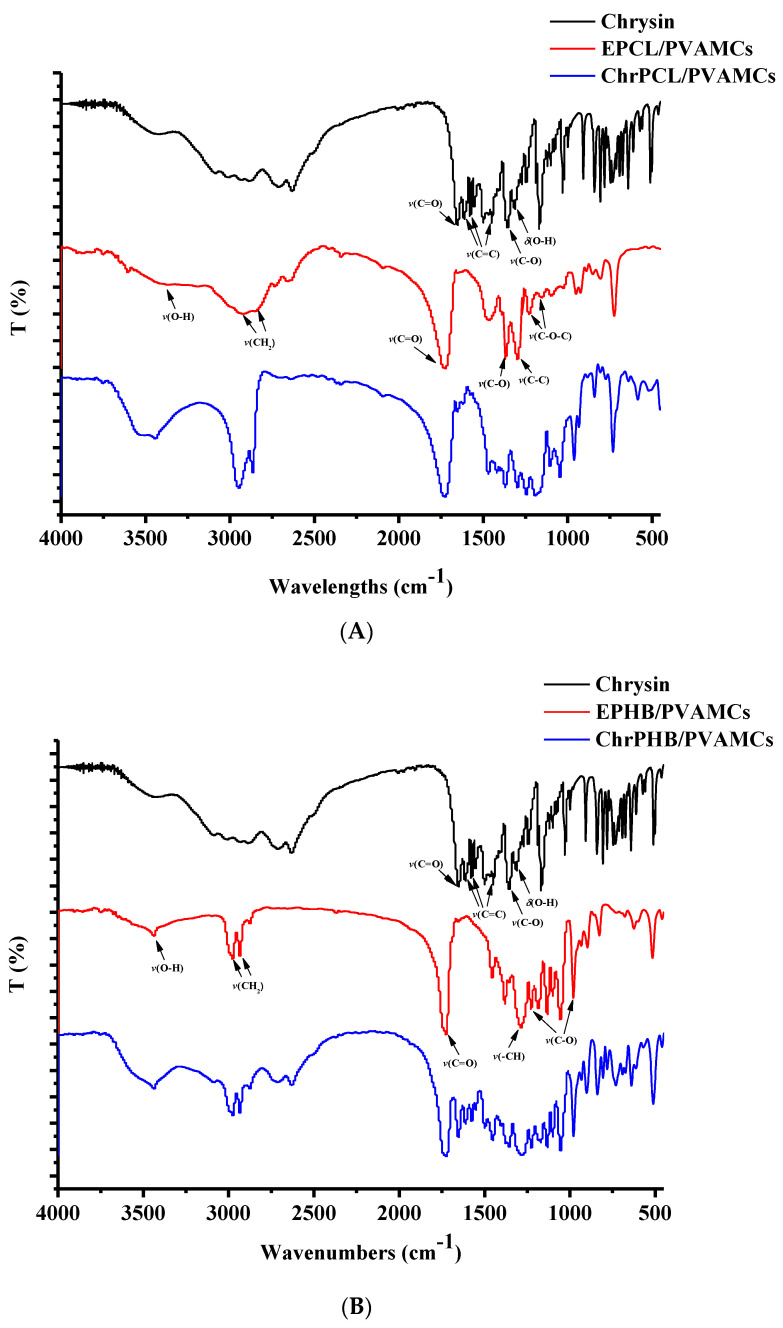
FT-IR spectra of (**A**) chrysin, EPCL/PVAMCs, ChrPCL/PVAMCs, and (**B**) chrysin, EPHB/PVAMCs, ChrPHB/PVAMCs.

FT-IR spectra of chrysin, EPCL/PVAMCs, EPHB/PVAMCs, ChrPCL/PVAMCs, and ChrPHB/PVAMCs are shown in Figure 3A,B. In the FT-IR spectrum of free chrysin the strong absorption band at 1650 cm^−1^ is assigned to the stretching vibrations of the carbonyl group *v*(C=O) coupled with the double band in the *γ*-benzopyrone ring [52,53]. Moreover, the absorption bands observed at 1450 cm^−1^, 1580 cm^−1^, and 1610 cm^−1^ are assigned to the *ν*(C=C) carbon vibrations in the *γ*-pyrone and benzene rings [52,53]. The absorption bands observed at 1360 cm^−1^ and at 1310 cm^−1^ are attributed to the coupled *ν*(C−O) and *δ*(O–H) vibrational modes, respectively [52,53]. In addition, the sharp absorption band at 1250 cm^−1^ is assigned to the *v*(C–O–C) stretching vibrations, whereas the broad band in the 3090–2640 cm^−1^ range is attributed to the *v*(C–H) and *v*(O–H) stretching vibrations [52,53].

In the spectrum of EPCL/PVAMCs the characteristic bands of the symmetric and asymmetric aliphatic *ν*(CH_2_) stretching vibrations of PCL can be observed at 2845 cm^−1^ and 2925 cm^−1^, respectively. The strong absorption band at 1730 cm^−1^ can be attributed to the carbonyl *ν*(C=O) stretching vibrations. The absorption bands of the PCL backbone *ν*(C–O) and *ν*(C–C) stretching vibrations are located at 1370 cm^−1^ and 1300 cm^−1^, respectively. Furthermore, the symmetric and asymmetric *ν*(C–O–C) vibrations appear at 1150 cm^−1^ and 1230 cm^−1^, respectively [54]. The broad absorption band in the 3590–3118 cm^−1^ range can be attributed to the *ν*(O–H) stretching vibrations of the PCL terminal hydroxyl groups and the PVA alcoholic moieties [55].

In the spectrum of EPHB/PVAMCs the two strong absorption bands observed at 1720 cm^−1^ and 1290 cm^−1^ are attributed to the carbonyl *ν*(C=O) stretching vibrations of the ester group and the *ν*(–CH) group, respectively. The absorption bands located in the range between 980 cm^−1^ and 1230 cm^−1^ can be assigned to the *ν*(C–O) stretching vibrations of the ester group. The absorption bands observed at 2980 cm^−1^ and 2930 cm^−1^ are indicative of the alkyl *ν*(–CH_3_) stretching vibrations, whereas the absorption band located at 1380 cm^−1^ is attributed to the *ν*(–CH_3_) symmetric bending vibrations. The band at 1460 cm^−1^ is assigned to the *ν*(–CH_2_) or *ν*(–CH_3_) asymmetric bending vibrations [56,57]. Moreover, the broad band at 3440 cm^−1^ can be attributed to the *v*(O–H) stretching vibrations of the PHB terminal hydroxyl groups and the PVA alcoholic moieties [55].

In the spectra of ChrPCL/PVAMCs and ChrPHB/PVAMCs all the important peaks of the biopolymers and chrysin are present. Variations in the IR peak intensity of both the host and guest molecules could be related to the intermolecular interactions induced by the encapsulation process [58].

### 3.3. FESEM Analyses

**Figure 4:**
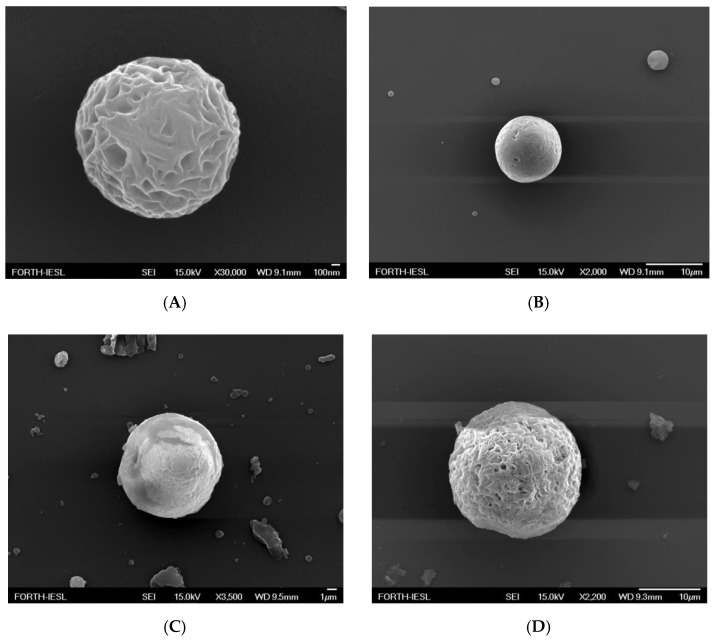
FESEM images of (**A**) EPCL/PVAMCs, scale bar: 100 nm, (**B**) ChrPCL/PVAMCs, scale bar: 10 µm, (**C**) EPHB/PVAMCs, scale bar: 1 µm, and (**D**) ChrPHB/PVAMCs, scale bar: 10 µm.

The morphological and structural characteristics of EPCL/PVAMCs, EPHB/PVAMCs, ChrPCL/PVAMCs, and ChrPHB/PVAMCs were examined by FESEM, and the results are presented in Figure 4. The EPCL/PVAMC sample (Figure 4A) consists of distinct globular microparticles with a highly wrinkled surface and sizes around 2 µm. FESEM images of the ChrPCL/PVAMC sample (Figure 4B) indicate the presence of scattered, spherical microparticles with a relative smooth surface. Their sizes range between 1.1 and 12.1 µm, showing a relatively wide size distribution. The EPHB/PVAMC sample (Figure 4C) consists of spherical, relatively wrinkled microparticles with sizes around 10.9 µm and scattered amorphous agglomerates, whereas FESEM images of the ChrPHB/PVAMC sample (Figure 4D) indicate the presence of globular microparticles with a highly spongy and wrinkled structure and sizes around 21.3 µm. The observed increase in the diameter of the chrysin-loaded species compared to their empty counterparts could be attributed to the encapsulation of chrysin molecules inside the polymeric structure, which induces the swelling of the microparticles. Literature reports on PVA-stabilized PCL microspheres loaded with flavonoid quercetin showed that all tested samples possessed a spherical morphology and wrinkled surface, but with large diameters, ranging between 61 and 171 µm. Furthermore, the increase of quercetin entrapment efficiency induced the size enlargement of the quercetin-loaded species compared to their empty counterparts [59]. Spherical morphology has also been observed for curcumin-loaded PHB/PVA micro-formulations exhibiting a semi smooth surface with pores of different sizes and mean diameters around 6.98 ± 1.89 µm [60].

### 3.4. Particle Size Analysis And Z-Potential

For comparative purposes, DLS and *z*-potential measurements were implemented to further determine the hydrodynamic mean diameter and surface charge of the EPCL/PVAMC, EPHB/PVAMC, ChrPCL/PVAMC, and ChrPHB/PVAMC samples. The mean hydrodynamic diameter and polydispersity index (PDI) of EPCL/PVAMCs and ChrPCL/PVAMCs were estimated to be 2.4 ± 1.3 µm (PDI = 2.03) and 11.8 ± 4.7 µm (PDI = 2.11), respectively. In the case of EPHB/PVAMCs and ChrPHB/PVAMCs, the values of the hydrodynamic mean diameter and PDI were found to be 10.4 ± 4.4 µm (PDI = 1.95) and 24.7 ± 8.5 µm (PDI = 1.93), respectively. The observed results clearly indicate that the encapsulation of chrysin significantly affects the size of the emerging MCs as also observed in other chrysin-loaded types of formulations reported in the literature [38,39,40,41,42,43]. Moreover, the relatively high PDI values could be attributed to the high PVA concentration during the synthetic procedure that resulted in the enhanced polydispersity of the produced MCs [59].

*Z*-potential measurements were conducted immediately after the determination of particle sizes. The *z*-potential values of EPCL/PVAMC, ChrPCL/PVAMC, EPHB/PVAMC, and ChrPHB/PVAMC samples were determined to be −16.2 ± 3.8 mV, −18.1 ± 4.1 mV, −14.1 ± 3.1 mV, and −16.3 ± 4.0 mV, respectively, presenting no significant differences between the empty and the chrysin-loaded MCs and confirming the highly negative surface charge of the produced microspheres, which promotes the formation of more stabilized and less aggregated MC dispersions due to the strong electrostatic repulsion forces between the microparticles [61]. It has been reported that the negatively charged surface of microparticles can potentially minimize non-specific binding with the cell membrane and, additionally, reduce aberrant protein binding. This prevents the activation of the immune system, thereby resulting in a prolonged circulatory half-life [62]. On the other hand, recent studies on novel synthetic drug nanocarriers based on zwitterionic biomimetic polymers and polypeptides have demonstrated that these materials, due to their structural characteristics, can be used not only for covalent modification with targeting ligands and biomolecules, but also for the prevention of nonspecific protein adsorption and maintainance of micelle stability in complex media, such as serum, thus providing long circulation lifetimes [63,64].

### 3.5. Entrapment Efficiency and Loading Capacity

The in situ entrapment efficiency of chrysin in the ChrPCL/PVAMCs and ChrPHB/PVAMCs was estimated to be 58.10% and 43.63%, whereas the loading capacity was found to be 3.79% and 15.85%, respectively. The obtained results are considered quite satisfactory and favorably comparable with those reported for other types of chrysin-loaded nanocarriers [38,39,40,41,43,65,66]. The observed high loading capacity of the ChrPHB/PVAMCs compared to that of the ChrPCL/PVAMC sample could potentially be attributed to the significantly porous structure of the PHB/PVA microparticles, as observed through FESEM, which might have promoted the encapsulation of chrysin in the interior of the pores [67], and the higher drug-to-polymer ratio applied during the synthetic procedure [58].

### 3.6. Release Study

**Figure 5:**
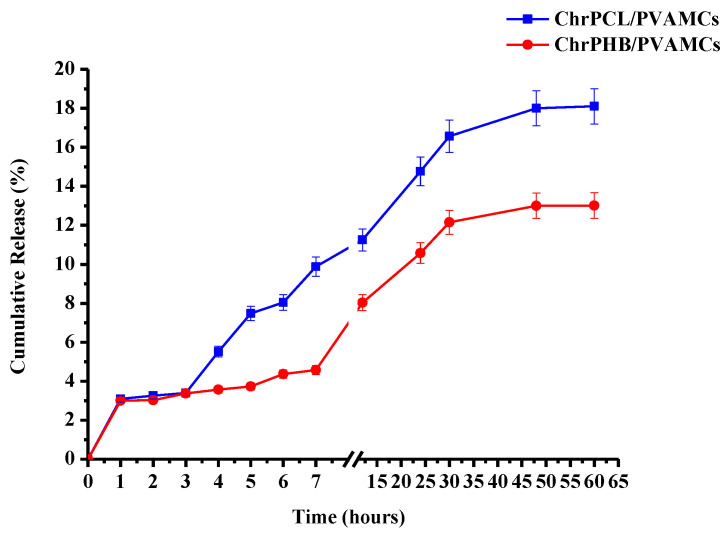
Cumulative release percentage of chrysin with regard to the total entrapped chrysin vs. time for ChrPCL/PVAMCs (blue line) and ChrPHB/PVAMCs (red line).

The release profile of the active agent (chrysin) is an important parameter, since it determines the pharmacokinetic behaviour of the chrysin-loaded MCs. In evaluating the release profile, two factors are taken into consideration: the total amount of chrysin released and the rate of release. Figure 5 presents the percentages of chrysin released with regard to the total entrapped chrysin versus time for both types of chrysin-loaded MCs. The average release percentage of chrysin from the ChrPCL/PVAMCs and ChrPHB/PVAMCs is 23.10% and 18.01%, respectively. By examining the release profile of the ChrPCL/PVAMC sample during the 60 h of study, it can be observed that up to the first 3 h the release rate is steady. Subsequently, a burst of chrysin release is observed which carries on up to 30 h, and then the process decelerates and the release rate is significantly decreased, reaching a plateau at 48 h. In the case of the ChrPHB/PVAMC sample, the chrysin release is relatively steady up to the first 7 h. Thereafter, the release rate increases and after 30 h begins to decelerate, approaching a plateau at 48 h. The observed low chrysin release percentages for both types of MCs can be attributed to the hydrophobic nature and the slow degradation rates of the employed biopolymers. Moreover, the relatively steady initial chrysin release rates, observed for both samples, can be due to their micro-sized dimensions. It is known that, in general, microparticles have a smaller surface area and higher porosity compared to nanoparticles. As a result, more drug molecules can be encapsulated into their pores than located near the particle surface which comes into direct contact with the aqueous medium, hence preventing their rapid diffusion [68].

Table 1 summarizes the results of the physico-chemical characterization of the produced empty and chrysin-loaded micro-formulations.

### 3.7. Breast Cancer Cell Viability after Exposure to Chrysin-Loaded MCs

**Figure 6:**
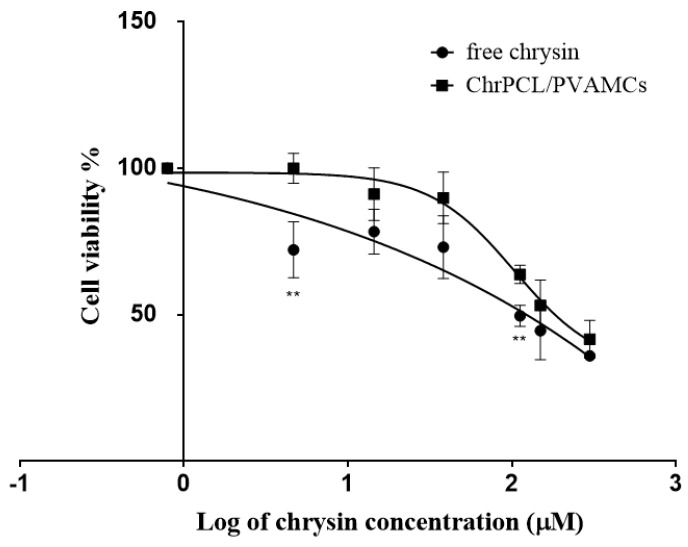
Cell viability (%) of MDA-MB-231 human breast cancer cells line exposed for 48 h to different concentrations (6.25, 12.5, 50, 100, 200 and 400 µg·mL^−1^) of ChrPCL/PVAMCs and to their corresponding equal molar concentrations of free chrysin. Cell viability was assessed using the MTT assay. The inhibitory concentration of chrysin for 50% viability (IC_50_) in MDA-MB-231 cells is 111.89 µM for free chrysin and 149.19 µM for ChrPCL/PVAMCs (encapsulated chrysin in the PCL/PVAMCs) ** *p* < 0.01.

**Figure 7:**
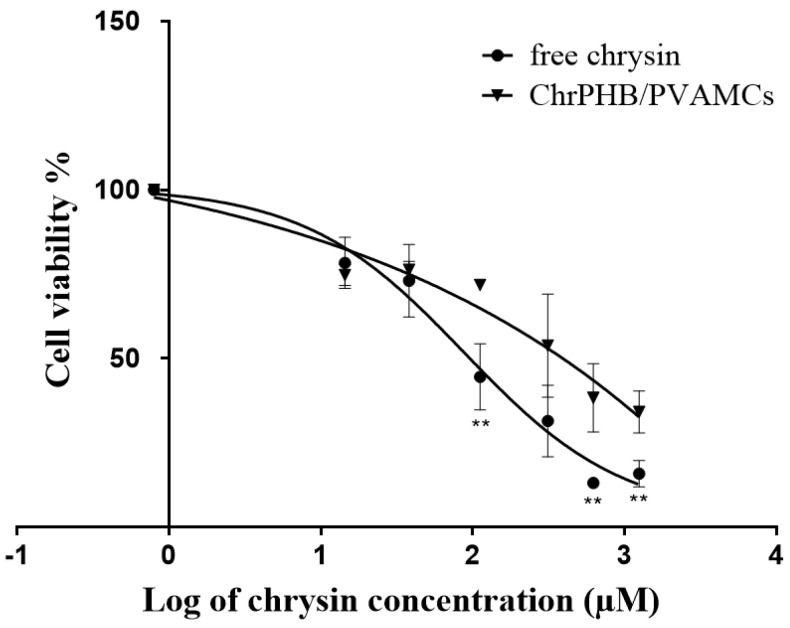
Cell viability (%) of MDA-MB-231 human breast cancer cells line exposed for 48 h to different concentrations (6.25, 12.5, 50, 100, 200 and 400 µg·mL^−1^) of ChrPHΒ/PVAMCs and to their corresponding equal molar concentrations of free chrysin. Cell viability was assessed using the MTT assay. The inhibitory concentration of chrysin for 50% viability (IC_50_) in MDA-MB-231 cells is 111.89 µM for free chrysin and 312.18 µM for ChrPHB/PVAMCs (encapsulated chrysin in the PHΒ/PVAMCs) ** *p* < 0.01.

The viability of the breast cancer cell line MDA-MB-231 was determined using the MTT assay after exposure to ChrPCL/PVAMCs or ChrPHB/PVAMCs. The cells were treated for 48 h with different concentrations of chrysin-loaded MCs (6.25, 12.5, 50, 100, 200, and 400 µg·mL^−1^). Due to the different loading capacity of chrysin in each type of MC, the corresponding molar amount of free chrysin was added as control. The results are shown in Figure 6 and Figure 7. The obtained results indicate that the micro-formulated chrysin inhibited the viability of cancer cells in a dose-dependent manner, but less so compared to free chrysin. Specifically, the ChrPCL/PVAMCs reached an IC_50_ value with an encapsulated chrysin content of 149.19 µM compared to that of free chrysin, which was 111.89 µM (Figure 6). The ChrPHB/PVAMCs reached an IC_50_ value with an encapsulated chrysin content of 312.18 µM (Figure 7). The higher IC_50_ values of the chrysin-loaded MCs compared to free chrysin can be attributed to the slow release rates and low release percentages of chrysin from both types of MCs due to the limited hydrophilicity and degradation rates of the employed biopolymers, which could potentially lead to the retarded inhibition of cell proliferation [69]. Moreover, as previously presented in the cumulative release diagram (Figure 5), the ChrPCL/PVAMCs have a higher release rate of chrysin, which explains the lower IC_50_ value compared to ChrPHB/PVAMCs. Furthermore, the EPCL/PVAMC and EPHB/PVAMC samples showed relatively low effect on cell viability (Supporting Information, Figure S1).

For comparative purposes, Table 2 presents the data reported in the literature on cytotoxic IC_50_ values of chrysin-loaded nano-formulations in several cancer cell lines. In most studied cases, nano-formulated chrysin showed lower IC_50_ values compared to free chrysin against various cancer cell lines, such as AGS, T47D, and MCF-7 [41,63,64], whereas in our case, both types of micro-formulated chrysin showed higher IC_50_ values, suggesting that the chrysin released from MCs was more slowly taken up by cells, potentially due to the slow release of chrysin, the limited degradation rates of the employed biopolymers [39], and the micro-dimensions of the produced carriers that affect cellular uptake to a certain degree. It is well-known that the surface area to volume ratio of microparticles is relatively low compared to that of nanoparticles [70]. As a result, cellular adherence on the surface of the MCs is limited, and thus cell attachment is hampered. Consequently, small numbers of cells can come into close contact with the released chrysin [71]. Moreover, another factor that can affect cytotoxicity, and conclusively the IC_50_ values, is based on the cellular exposure time to the nano- or micro-formulated chrysin reflecting more or less on the cellular growth inhibition, as is obvious in Table 2. Indicatively, it has been shown that PLGA/PVA chrysin nano-formulations ameliorated the delivery of chrysin through a higher absorption by cells and enhanced its effectiveness on cell growth inhibition [38]. In general, it should be noted that, compared to those in the literature, the tested cell line in this study (MDA-MB-231) is highly aggressive, with limited treatment options, invasive, and poorly differentiated triple-negative breast cancer (TNBC) cell line, as it lacks estrogen receptor (ER) and progesterone receptor (PR) expression, as well as HER2 (human epidermal growth factor receptor 2) amplification. However, despite all these factors, the MCs under investigation exhibited sufficient cytotoxicity against this aggressive breast cancer cell line.

### 3.8. Effect of Chrysin-Loaded MCs on Blood Profile Analysis

**Figure 8:**
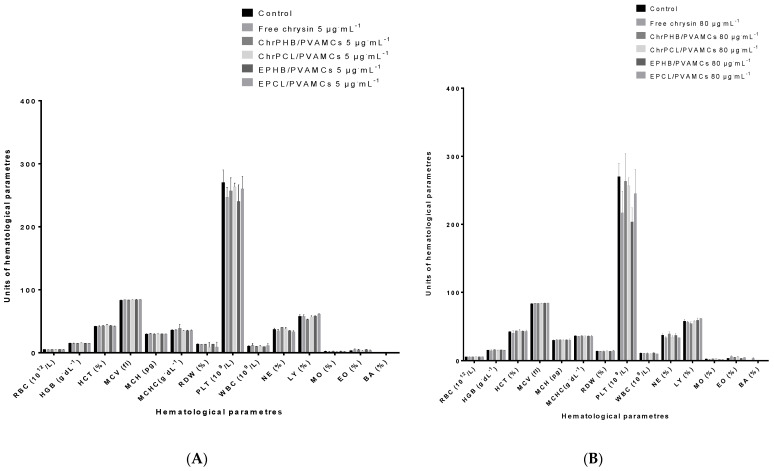
Hematological parameters after the treatment of human blood samples with two concentrations, (**A**) 5 µg·mL^−1^ and (**B**) 80 µg·mL^−1^, of free chrysin, ChrPCL/PVAMCs, or ChrPHB/PVAMCs and their empty counterparts. RBC: red blood cells (10^12^/L); HGB: hemoglobin (g·dL^−1^); HCT: hematocrit (%); MCV (fl); MCH (pg); MCHC (g·dL^−1^); RDW (%); PLT (10^9^/L); WBC (10^9^/L); NE (%); LY (%), MO (%), EO (%), and BA (%).

The collective measurements of the hematological parameters after human blood exposure to 5, 80, and 200 µg·mL^−1^ of free chrysin and chrysin-loaded and empty MCs are shown in Figure 8A,B and the Supporting information, Figure S2, respectively. The observed values of almost all the hematological parameters of the blood samples that were treated with plain chrysin, ChrPCL/PVAMCs, or ChrPHB/PVAMCs and their empty counterparts at 37 °C for 1 h did not display significant deviation compared to the negative control sample, indicating no concentration-dependent alteration. These parameters include RBCs, HGB, HCT, MCV, MCH, MCHC, RDW, WBCs, NE, LY, MO, EO, and BA. However, a significant decrease in PLT values was observed between the negative control sample and pure chrysin, indicating a concentration-dependent inhibition, therefore confirming the antiplatelet activity of chrysin [72]. Moreover, a small decrease in PLT values was also observed after treatment with the EPHB/PVAMCs in both concentrations tested, also confirming the inhibitory effect of PHB on isolated platelets [73]. Thrombocytopenia is the result of a reduction in the number of blood platelets and it can be a side effect of taking certain medications. As each platelet lives only about 10 days, our body normally renews our platelet supply continually by producing new platelets in our bone marrow [74]. Collectively, the obtained results clearly point out the sufficient blood compatibility of the prepared chrysin-loaded MCs at low and high concentrations and their prospect for potential use in several bio-applications, such as tumor therapy.

### 3.9. Hemolysis

**Figure 9:**
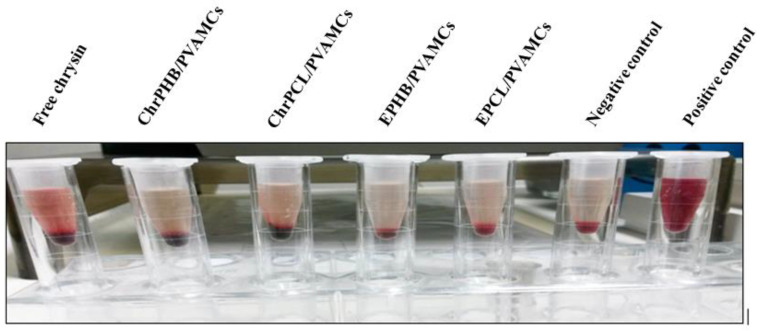
Micrograph of human RBCs showing the degree of hemolysis after incubation with 500 µg·mL^−1^ of free chrysin, ChrPHB/PVAMCs, ChrPCL/PVAMCs, EPHB/PVAMCs, and EPCL/PVAMCs.

It is well established that nanoparticles possess properties that can induce hemolysis and decrease the efficiency of anticancer drugs in vitro [75]. In the effort to evaluate the hemocompatibility of encapsulated chrysin in MCs, a hemolysis study was performed using chrysin as positive control and PBS as negative control. Hemolysis is the rupturing of RBCs and the subsequent release of hemoglobin upon destruction of the red cell membrane [76]. The quantitative determination of the released hemoglobin can provide evidence on the potential damage to RBCs after MC administration; and this can serve as a viable indicator of MC toxicity under in vivo conditions [38]. Based on the criterion established by the American Society for Testing and Material (ASTM) E2524—08(2013) active standard [77], a test method for the analysis of the hemolytic properties of nanoparticles, it has been reported that a percentage of induced hemolysis greater than 5% indicates a damage on RBCs [78]. In our case, the obtained results from the hemolysis assay showed that ChrPCL/PVAMCs, ChrPHB/PVAMCs, and their empty counterparts displayed great compatibility with RBCs, as their hemolytic percentages were significantly low (up to 2%) at various concentrations ranging between 5 and 500 µg·mL^−1^ (Table 3). On the other hand, free chrysin, which was diluted in 5% DMSO so as to enhance its solubility, displayed a hemolytic activity higher than 5%, but only in concentrations between 100 and 500 µg·mL^−1^, indicating a relative RBC damage. For comparative purposes, we pose that chrysin-loaded PLGA-PVA nanoparticles exhibited a hemolysis percentage within the admissible limit of less than 3% for very low concentrations of nanoparticles (5–20 µg·mL^−1^), whereas concentrations in the range between 40 and 80 µg·mL^−1^ induced a hemolysis percentage lower than 5%, and yet lower than that of free chrysin at the same concentrations [38]. It should also be noted that different blood groups of the ABO system have a specific antigen which endows them with different biochemical properties, and hence they can show different hemolytic activity [79]. The collective micrographs of human RBCs resulting from the hemolysis assay, after exposure to a high concentration (500 µg·mL^−1^) of free chrysin, ChrPHB/PVAMCs, ChrPCL/PVAMCs, EPHB/PVAMCs, and EPCL/PVAMCs, are visualized in Figure 9. It can be concluded that the hemoglobin release from RBCs is obvious after exposure to free chrysin, whereas in the case of the empty and chrysin-loaded MCs no hemoglobin release is observed. The hemolytic data are consistent with those of the hematological parameters proving that the employed MCs display sufficient hemocompatibility.

## 4. Conclusions

In the pursuit of the development of bioavailable, long-life, and stable microcarriers of natural products, such as bioflavonoids, novel poly(*ε*-caprolactone), and poly(3-hydroxybutyrate) microcarriers of flavonoid chrysin, were synthesized, physico-chemically characterized, and biologically evaluated for their hemolytic capacity and degree of toxicity against the epithelial human breast cancer cell line MDA-MB-231. The bioavailable and biocompatible nature of the emerged micro-formulations, their physico-chemical and morphological features, and their sufficient human blood compatibility and cytotoxic activity toward cancer cells indicate the ability of MCs to function as efficient delivery vehicles of bioactive flavonoids, and render them ideal micro-platforms for further therapeutic applications against cancer and common blood diseases.

## Figures and Tables

**Table 1:** Physico-chemical characterization data of the produced empty and chrysin-loaded micro-formulations.

Micro-Formulation	d_FESEM_^a^ (µm)	d_DLS_^b^ (µm)	PDI	*Z*-Potential (mV)	Entrapment Efficiency (%)	Loading Capacity (%)	In Vitro Release (%)
EPCL/PVAMCs	2	2.4 ± 1.3	2.03	−16.2 ± 3.8	-	-	-
EPHB/PVAMCs	10.9	10.4 ± 4.4	1.95	−14.1 ± 3.1	-	-	-
ChrPCL/PVAMCs	1.1–12.1	11.8 ± 4.7	2.11	−18.1 ± 4.1	58.10	3.79	23.10
ChrPHB/PVAMCs	21.3	24.7 ± 8.5	1.93	−16.3 ± 4.0	43.63	15.85	18.01

^a^ Diameter observed via FESEM. ^b^ Hydrodynamic mean diameter measured via DLS.

**Table 2:** Data reported in the literature on cytotoxic IC_50_ values of chrysin-loaded nano-formulations.

Type of Chrysin-Loaded Nano-Formulation	Cell Line	Treatment Duration (Hours)	IC_50_	References
Methoxy PEG-β-PCL nanoparticles	A549 non-small-cell lung cancer	48	2.5 µM	[39]
PLGA-PEG-PLGA nanoparticles	AGS gastric cancer	24, 48, 72	58.2, 44.2, 36.8 µM	[41]
PCL-PEG-PCL nanoparticles	T47D breast cancer	24, 48, 72	2, 10, 10 µM	[63]
PLGA-PEG nanoparticles	T47D breast cancer	24, 48, 72	40.19, 35.75, 31.28 µM	[64]
	MCF-7 breast cancer		66.41, 56.80, 42.54 µM	

**Table 3:** Concentration-dependent hemolytic activity of free chrysin, ChrPHB/PVAMCs, ChrPCL/PVAMCs, EPHB/PVAMCs, and EPCL/PVAMCs.

Concentration (µg·mL^−1^)	Free Chrysin *	ChrPHB/PVAMCs	ChrPCL/PVAMCs	EPHB/PVAMCs	EPCL/PVAMCs
Percentage of Hemolysis (%)					
5	1.2	0.2	0.1	0.03	0.02
20	2.1	0.3	0.1	0.04	0.03
40	2.7	0.5	0.3	0.03	0.03
60	3.0	0.6	0.5	0.05	0.6
80	3.5	0.7	0.7	0.07	0.06
100	6.8	1.1	1.0	0.1	0.1
200	7.0	1.4	1.2	0.3	0.3
300	7.3	1.4	1.3	0.5	0.6
400	7.9	1.6	1.5	0.6	0.6
500	8.2	2.0	1.8	0.6	1.0

* Chrysin solution was prepared in DMSO (5%).

## Data Availability

All relevant data are included in the article and/or its Supplementary Information files.

## Supplementary Materials

The following are available online at https://www.mdpi.com/1999-4923/13/1/109/s1, Figure S1: MTT cytotoxicity assay for the empty MCs. Figure S2: Hematological parameters after the treatment of human blood samples with 200 μg·mL^−1^ of free chrysin, ChrPCL/PVAMCs or ChrPHB/PVAMCs and their empty counterparts. Table S1: Percentages of the insoluble solid chrysin-loaded MCs.

## Abbreviations

GIT	Gastrointestinal tract
PHAs	Polyhydroxyalkanoates
PCLs	Poly(*ε*-caprolactones)
PHB	Poly(3-hydroxybutyric acid)
PLGA	Poly(lactide-*co*-glycolide)
PLA	Polylactate
PGA	Polyglycolate
PVA	Poly(vinyl alcohol)
Chr	Chrysin
MCs	Microcarriers
EPCL/PVAMCs	Empty PVA-stabilized PCL microcarriers
EPHB/PVAMCs	Empty PVA-stabilized PHB microcarriers
ChrPCL/PVAMCs	Chrysin-loaded PVA-stabilized PCL microcarriers
ChrPHB/PVAMCs	Chrysin-loaded PVA-stabilized PHB microcarriers
DPPH	2,2-diphenyl-1-picrylhydrazyl
NaOH	Sodium hydroxide
PBS	Phosphate buffered saline
IMBB	Institute of Molecular Biology and Biotechnology
MTT	3-(4,5-dimethylthiazol-2-yl)-2,5-diphenyl tetrazolium bromide
DMSO	Dimethyl sulfoxide
FT-IR	Fourier-transform infrared
FESEM	Field emission scanning electron microscopy
DLS	Dynamic Light Scattering
SD	Standard deviation
HPLC	High Performance Liquid Chromatography
DAD	Diode array detector
O/W	Oil-in-water
DMEM	Dulbecco′s Modified Eagle′s—Medium
FBS	Fetal Bovine Serum
RBCs	Red blood cells
HGB	Hemoglobin
HCT	Hematocrit
MCV	Mean corpuscular volume
fl	Femtoliters
MCH	Mean corpuscular hemoglobin
MCHC	Mean corpuscular hemoglobin concentration
RDW	Red cell distribution width
WBCs	White blood cells
NE	Neutrophils
LY	Lymphocytes
MO	Monocytes
EO	Eosinophils
BA	Basophils
PLTs	Platelets
EDTA	Ethylenediamine tetraacetic acid
TNBC	Triple-negative breast cancer
ER	Estrogen receptor
PR	Progesterone receptor
HER2	Human epidermal growth factor receptor 2
ASTM	American Society for Testing and Material
