# Supplementary material for: Evaluation of the Hemocompatibility and Anticancer Potential of Poly(ε-Caprolactone) and Poly(3-Hydroxybutyrate) Microcarriers with Encapsulated Chrysin
Source: Pharmaceutics. 2021 Jan 16;13(1):109. doi: 10.3390/pharmaceutics13010109 (PMC7831015; doi:10.3390/pharmaceutics13010109)
Supplement: Supplementary file 1 [file pharmaceutics-13-00109-s001.pdf]

Supplementary Materials: Evaluation of the Hemocompatibility and Anticancer Potential of Poly(ε-Caprolactone) and Poly(3-Hydroxybutyrate) Microcarriers with Encapsulated Chrysin

Eleftherios Halevas *, Chrysoula Kokotidou, Elda Zaimai, Alexandra Moschona, Efstratios Lialiaris, Anna Mitraki, Theodore Lialiaris and Anastasia Pantazaki *

Table S1. Percentages of the insoluble solid chrysin-loaded MCs.

Time (hours)	Insoluble ChrPCL/PVAMCs (%)	Insoluble ChrPHB/PVAMCs (%)
0	100	100
1	96.90	96.99
2	96.74	96.97
3	96.61	96.62
4	89.48	96.43
5	87.53	96.27
6	86.96	95.63
7	85.12	95.42
12	83.75	86.97
24	80.23	84.43
30	78.43	82.85
48	77.00	82.00
60	76.90	81.99

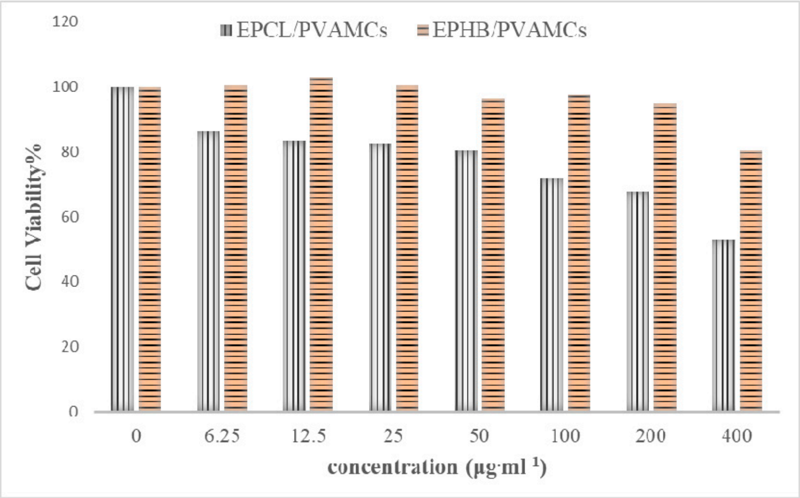

Figure S1. MTT cytotoxicity assay for the empty MCs. MDA-MB-231 cells were treated with increased concentrations (0–400 μg·mL^{-1}) of EPCL/PVAMCs and EPHB/PVAMCs for 48 h. The EPHB/PVAMCs exhibited insignificant cytotoxicity with the cell viability remaining above 80%, even at the highest MC concentration used, whereas the EPCL/PVAMCs, at concentrations above 200 μg·mL^{-1}, showed relative cytotoxicity by decreasing cell viability to 50–60%.

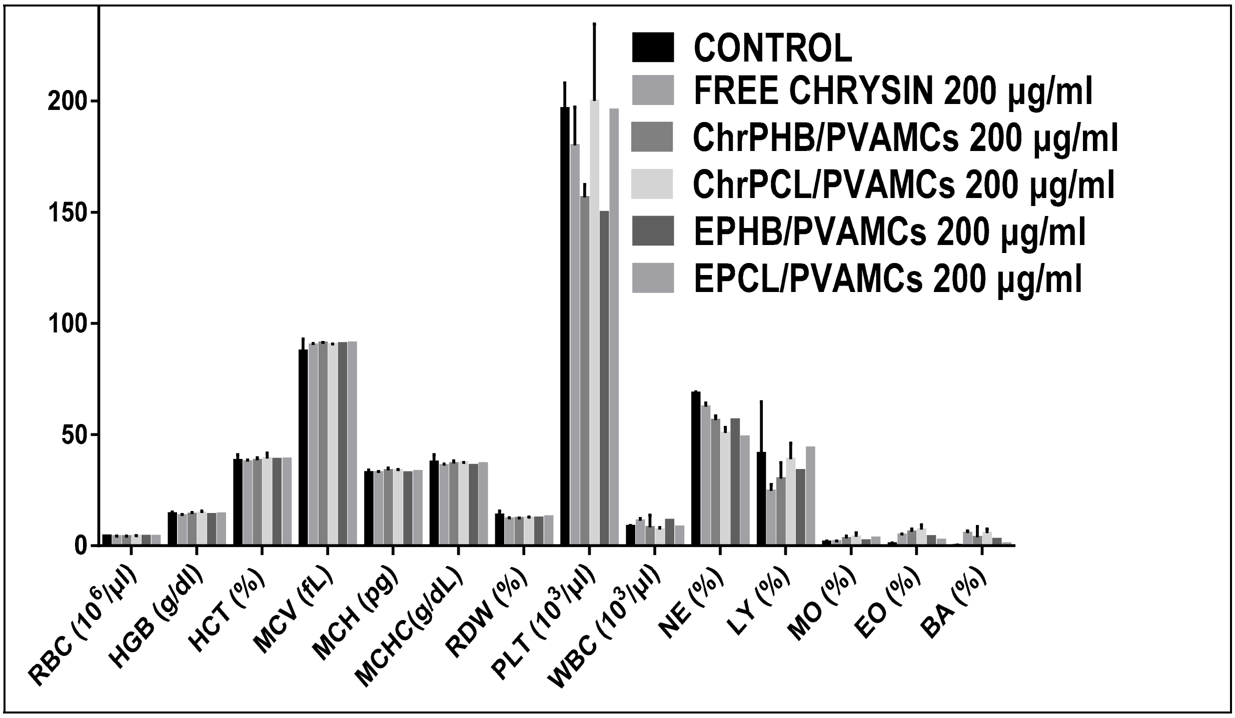

Figure S2. Hematological parameters after the treatment of human blood samples with 200 µg·mL^{-1} of free chrysin, ChrPCL/PVAMCs or ChrPHB/PVAMCs and their empty counterparts. RBC: red blood cells (10^{12}/L); HGB: haemoglobin (g·dL^{-1}); HCT: haematocrit (%); MCV (fl); MCH (pg); MCHC (g·dL^{-1}); RDW (%); PLT (10^9/L); WBC (10^9/L); NE (%); LY (%), MO (%), EO (%), and BA (%).
